# Mining candidate genes associated with powdery mildew resistance in cucumber via super-BSA by specific length amplified fragment (SLAF) sequencing

**DOI:** 10.1186/s12864-015-2041-z

**Published:** 2015-12-14

**Authors:** Peng Zhang, Yuqiang Zhu, Lili Wang, Liping Chen, Shengjun Zhou

**Affiliations:** Institute of Vegetable, Zhejiang Academy of Agriculture Sciences, Hangzhou, 310021 China

**Keywords:** SLAF-seq, Super-BSA, Powdery mildew resistance, Cucumber

## Abstract

**Background:**

Powdery mildew (PM) is the most common fungal disease of cucumber and other cucurbit crops, while breeding the PM-resistant materials is the effective way to defense this disease, and the recent development of modern genetics and genomics make us aware of that studying the resistance genes is the essential way to breed the PM high-resistance plant. With the ever increasing throughput of next-generation sequencing (NGS), the development of specific length amplified fragment sequencing (SLAF-seq) as a high-resolution strategy for large-scale *de novo* SNP discovery is gradually applied for functional gene mining. Here we combined the bulked segregant analysis (BSA) with SLAF-seq to identify candidate genes associated with PM resistance in cucumber.

**Methods:**

A segregating population comprising 251 F2 individuals was developed using H136 (female parent) as susceptible parent and BK2 (male parent) as resistance donor. After PMR test, total genomic DNA was prepared from each plant. Systemic genomic analysis of the GC content, repeat sequence, etc. was carried out by prediction software SLAF_Predict to establish condition to ensure the uniformity and density of the molecular markers. After samples were gel purified, SLAFs were generated at Biomarker Technologies Corporation in Beijing. Based on SLAF tags and the PMR test result, the hot region were annotated.

**Results:**

A total of 73,100 high-quality SLAF tags with an average depth of 99.11× were sequenced. Among these, 5,355 polymorphic tags were identified with a polymorphism rate of 7.34 %, including 7.09 % SNPs and other polymorphism types. Finally, 140 associated SLAFs were identified, and two main Hot Regions were detected on chromosome 1 and 6, which contained five genes invovled in defense response, toxin metabolism, cell stress response, and injury response in cucumber.

**Conclusions:**

Associated markers identified by super-BSA in this study, could not only speed up the study of the PMR genes, but also provide a feasible solution for breeding the marker-assisted PMR cucumber. Moreover, this study could also be extended to any other species with reference genome.

## Background

Powdery mildew (PM), mainly caused by *Podosphaera fusca* (Fr.) Braun & Shishkoff, which affects a wide range of plants, and is the most common fungal disease of cucumber (*Cucumis sativus* L.) and other cucurbit crops in both greenhouse and field [1]. P. xanthii is predominant in China. It can provoke a variety of symptoms after infection such as talcum-like, whitish and powdery fungal growth [2, 3]. Although biological controls and transgenic have some effect, breeding the PM-resistant plant was still the effective way by now.

In China, breeding of excellent resistance cucumbers began in the mid-1950s. From the 1970s, a series of high quality disease-resistant varieties had been cultivated, e.g. Jinyan II, IV, Jinza I, II, III, IV etc. [4]. Recent development of modern genetics and genomics make us aware of that studying the resistance genes is the essential in order to breed the PM high-resistance plant. Since 1940s, PM resistance (PMR) and its inheritance have been declared in a number of cucumber lines. Smith reported that PMR in the cucumber cultivar ‘Puerto Rico 37’ was due to multiple recessive factors [5]. While PMR in PI 197087 may be controlled by 1–2 major and 1–2 minor genes [6]. Fujieda and Akiya identified a single recessive gene underlying the PMR in ‘Natsufushinari’ (PI 279465 from Japan) [7], whereas Kooistra proposed three recessive genes for PMR in cucumbers: two from Natsufushinari and one from PI 200815 or PI 200818 [5]. Shanmugasundaram et al. took the lead in studying PMR of differentiate hypocotyl and leaf in cucumbers, and they suggested a recessive gene for hypocotyl resistance that played an important role in overall performance of PMR. [8] Classical genetic analysis found that PMR in cucumber was linked with the D locus for dull fruit color [9–11] which has been mapped in cucumber chromosome 5 [12].

Since no single gene has been identified, the quantitative trait loci (QTL) mapping strategy provided effective tools for molecular dissection of PMR in cucumbers [13–18]. For example, using 97 recombinant inbred lines (RIL) and 154 markers, six temperature-dependent QTL were detected in four linkage groups (LGs) underlying PMR. [13] With 130 F_2:3_ lines in two environments, five QTL in three LGs were considered to be responsible for PMR originated from a European greenhouse type cucumber line S06 [16]. In yet another study, using the Two F_2_ populations derived from Ano2 × Hami413 and Ano2 × Queen, a domain gene Pm-AN was mapped between two codominant markers RPW and MRGH63B in LG V [19]. More recently, a 3-year QTL mapping study of PMR was conducted with 132 F_2:3_ families with 240 SSR markers, as a result, six genomic regions in four chromosomes harboring QTL for PMR in WI 2757 were identified. The two major QTL, pm5.1 and pm5.2 were located in chromosome 5 with the phenotypic variations of 21.0 and 74.5 % respectively [20]. Nearly the same time, a cucumber genetic linkage map consisted of 296 markers was constructed based on a population of 111 RIL, and Four QTL underlying PMR (pm3.1, pm5.1, pm5.2 and pm5.3) were successfully validated [21]. Although these studies have provided insights into the genetic control of PMR in cucumbers, a clear picture continues to be lacking. The numbers and locations of QTL identified in these studies are inconsistent, which may be due to the sources of PMR, the methods of bioassay, and environmental conditions used by different researchers. In addition, molecular markers identified from these studies were not breeder friendly. It is particularly important that the resolution of genetic map is not high enough for practical use in marker-assisted selection, not to mention fine mapping or cloning of the major-effect QTL.

Single Nucleotide Polymorphisms (SNPs) are currently the more appropriate choice for genetic analysis based on sequencing. With the ever increasing throughput of next-generation sequencing (NGS), *de novo* and reference-based SNP discovery has been demonstrated in several species and is gradually becoming the new method for functional gene mining [22–24]. Recently, Sun et al. reported the development of specific length amplified fragment sequencing (SLAF-seq) as a high-resolution strategy for large-scale de novo SNP discovery and genotyping. It allowed researchers to design the experimental system through bioinformatics and screen for fragments of a specific length from the constructed SLAF-seq library [25]. The massive sequences were then achieved and analyzed using SLAF_Poly.pl. (Biomarker, Beijing, China). After sequence the alignment through BLAT [26], a large number of specific fragments were selected for specific molecular markers development. SLAF-seq technology has several obvious advantages, such as high throughput, high accuracy, low cost and short cycle, which enable its results to be directly used for molecular markers development. This technology has been made available for haplotype mapping, genetic mapping, linkage mapping, and polymorphism mapping. It can also provide an important basis for molecular breeding, system evolution and germplasm resource identification [25].

In this study, we employed the recently developed SLAF-seq approach to achieve the first mass rapid discovery of SNP and insertion-deletion (InDel) markers for cucumber. Using these newly developed markers, a super-BSA (bulked segregant analysis) was performed to identify candidate genes associated with PMR. Our approach for candidate gene identification can be extended to any other species with reference genome.

## Methods

### Plant material and disease inoculation

A segregating population comprising 251 F_2_ individuals was developed using H136 (female parent) as susceptible parent and BK2 (male parent) as resistance donor. Both parents and segregating population were sown in Yangdu experimental base of Zhejiang Academy of Agriculture Sciences.

PMR test was conducted as described previously with some modifications. Briefly, both parents and segregating population were sown in soil on a plastic tray in the intelligent greenhouse. When seeding grows to one-leaf stage, they were inoculated with the pathogen by a spray of spore suspension (1 × 10^4^ ~ 1 × 10^6^ spores per ml). This was followed by incubation under 26 °C with a 16 h photoperiod for 15 days. Furthermore, infection of leaf was record every three days after inoculation [26].

The disease index (DI) was classified into the following ten categories based on visual infection of the leaf: 0 = no or almost no symptom; 1 = faint spot; 2–3 = thin mat of mildew; 4–5 = thick mat of mildew; 6–7 = very thick mat of mildew; 8–9 = whole leaf surface coated with mildew. DI from the average value of three independent tests on each line was used for subsequent analysis [54].

### DNA extraction and digestion design

Two parents (RP and SP) along with two phenotypically contrasting bulks, one resistant and one susceptible to PM and each comprising 50 F_2_ plants were generated. Leaves in two-leaf stage were collected, frozen in liquid nitrogen, and used for DNA extraction. Total genomic DNA was prepared from each plant according to the cetyltrimethylammonium bromide (CTAB) method [55]. DNA concentration and quality were estimated with an ND-1000 spectrophotometer (NanoDrop, Wilmington, DE, USA) and by electrophoresis in 0.8 % agarose gels with a lambda DNA standard. Then, the resistant and susceptible bulks were mixed respectively to form two samples called resistant-mix (RM) and susceptible-mix (SM). Systemic genomic analysis of the GC content, repeat sequence, etc. was carried out by prediction software SLAF_Predict to establish condition to ensure the uniformity and density of the molecular markers.

### SLAF library construction and high–throughput sequencing

The procedure was performed as described by Sun et al*.* with minor modifications. Briefly, based on the result of the software prediction, the SLAF library was constructed as following. About 500 ng genomic DNA of four samples (RP, SP, RM and SM) was first incubated at 37 °C with 0.6 U *MseI* [NEB, Hitchin, Herts, UK], T4 DNA ligase (NEB), ATP (NEB), and *MseI* adapter. Restriction/ligation reactions were heat-inactivated at 65 °C for 1 h and digested with *Hae III* and *Bfa I* restriction enzymes at 37 °C for 3 h. Then, polymerase chain reactions (PCR) were carried out in the reaction solutions containing the diluted restriction/ ligation samples, dNTP, *Taq* DNA polymerase (NEB), and *MseI*-primer containing barcode 1. PCR products were purified using an E.Z.N.A.® Cycle Pure Kit (Omega Bio-Tek, Norcross, GA, USA) and pooled. Pooled samples were incubated at 37 °C with *MseI*, T4 DNA ligase, ATP, and Solexa adapter, purified using a Quick Spin column (Qiagen, Hilden, Germany), and run on a 2 % agarose gel. Fragments of 230–250 bp (with indices and adaptors) were isolated using a Gel Extraction Kit (Qiagen) and subjected to PCR amplification with Phusion Master Mix (NEB) and Solexa Amplification primer mix (Illumina, Inc., San Diego, CA, USA) to add barcode 2 according to the Illumina sample preparation guide. After samples were gel purified, DNA fragments (SLAFs) of 230–250 bp were excised and diluted for pair-end sequencing on an Illumina GAIIx sequencing platform (Illumina, Inc; San Diego, CA, U.S.) at Biomarker Technologies Corporation in Beijing (http://www.biomarker.com.cn/ english/). Real-time monitoring was performed for each cycle during sequencing, the ratio of high quality reads with quality scores greater than Q_20_ (means a quality score of 20, indicating a 1 % chance of an error, and thus 99 % confidence) in the raw reads and guanine-cytosine (GC) content were calculated for quality control [25].

### SLAF-seq data grouping and genotype definition

After sequencing, the raw reads were screened via a perl script SLAF_Poly.pl to filter out the low quality data with a threshold of Valid-Data ≥ 50,000 and Depth ≥ 5×. All SLAF pair-end reads with clear index information were clustered based on sequence similarity by BLAT (tileSize = 11, stepSize = 11, minScore = 30). Sequences with an identity of over 90 % were grouped together in one SLAF locus. Then the error correction was implemented as described by Sun et al*.* to produce high quality data. At the same time, these data were assembled on the cucumber reference genome (http://www.icugi.org/cgi-bin/ICuGI/genome/home.cgi?organism=cucumber&ver=2).by BLAT (tileSize = 11, stepSize = 11, minScore = 30). Those reads clustered by sequence similarity could form a group, in which only those reads with a higher depth could be considered as a potential genotype whereas the lower reads would be corrected or removed as described by Sun et al*.*.

### Polymorphism and association analysis

Because cucumber is a diploid species, one locus contains at most four SLAF tags. Whereas the materials used throughout this study were two cultivars and F_2_ individuals derived from their crossing. Theoretically, there could be but two genotypes for one locus, as a result SLAFs with two tags were identified as polymorphic SLAFs and considered to be potential markers. The association analysis was conducted by comparing the depth of different polymorphic SLAF tags in RM and SM. Before this, the depth of each tag in RM and SM was standardized according to that of the parents. Then the Difference Ratio (DR value) of each locus in the two F2 bulks was calculated respectively, and *Ratio*_*rm* > =1 && *Ratio*_*sm* > =3 was considered as the determination criterion for associated SLAFs. The calculation method of DR value was described below:

$$ \begin{array}{l} Ratio\_rm=\left\{\begin{array}{l}RP\_rm/SP\_rm,SP\_rm>0\\ {}1000,SP\_rm=0\end{array}\right.\\ {} Ratio\_sm=\left\{\begin{array}{l}SP\_sm/RP\_sm,RP\_sm>0\\ {}1000,RP\_sm=0\end{array}\right.\\ {}\end{array} $$

Where *“RP*_*rm”* and *“SP_rm”* denoted the depth of SLAFs derived from RP and SP respectively in RM, and “*RP_sm”* and “*SP_sm”* denoted the depth of SLAFs derived from RP and SP respectively in SM.

### Hot region annotation and transcript abundance analysis

A list of genes within the hot regions was generated using the genome annotation data, and these genes were used to query the GO annotation on the website of gene ontology to identify putative resistance related genes for further analysis.

### Availability of supporting data

All the supporting data are included as additional files.

## Results

### Analysis of SLAF-seq data and SLAF tags development

**Fig. 1:**
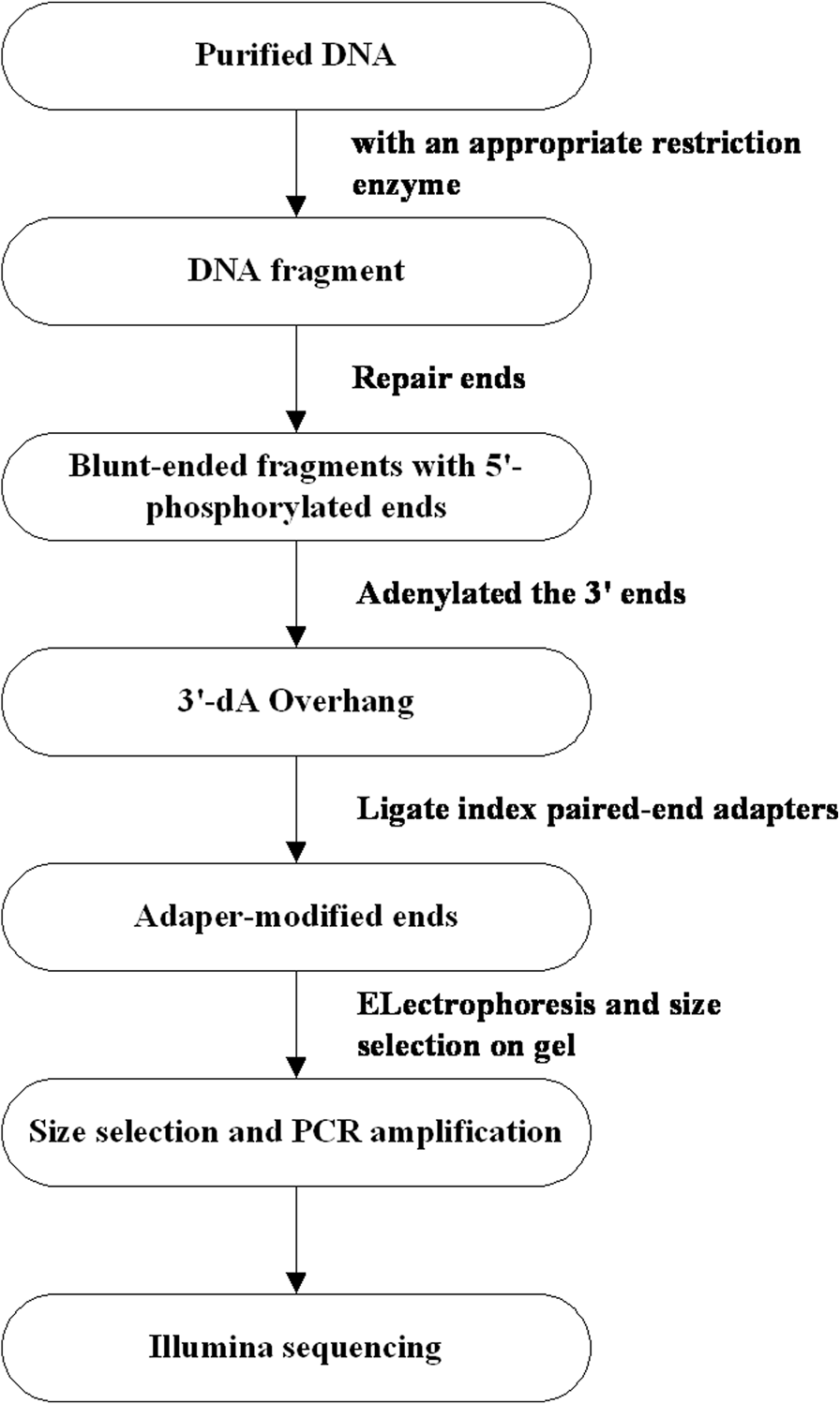
Flowchart of SLAF-SeqD

DNA samples from the two parents (H136 and BK2)along with the two separate bulks from the F_2_ population derived from H136 × BK2 was subjected to SLAF-Seq (Fig. 1). After SLAF library construction and high-throughput sequencing, a total of 1.26 Gb of data containing 16,977,114 pair-end reads were obtained with each read being ~80 bp in length and guanine-cytosine (GC) content was 38.96 % (Table 1).

**Table 1:** Reads distribution and GC percentage

Sample	BMK-ID	Read length(bp)	Read number	GC percentage
SP	BK2	80	3,293,311	39.35 %
RP	H136	80	4,517,307	38.83 %
SM	Susceptible Pool	80	5,073,477	39.03 %
RM	Resistant Pool	80	4,093,019	38.61 %

Of these high-quality data, ~251 Mb was from Resistant Parent (RP) with 3,293,311 reads, and ~345 Mb were from the Susceptible Parent (SP) with 4,517,307 reads. Read numbers for the Susceptible Mix (SM) and Resistant Mix (RM) were 5,073,477 and 4,093,019 respectively with an average of 4,583,248 (Table 1).

**Fig. 2:**
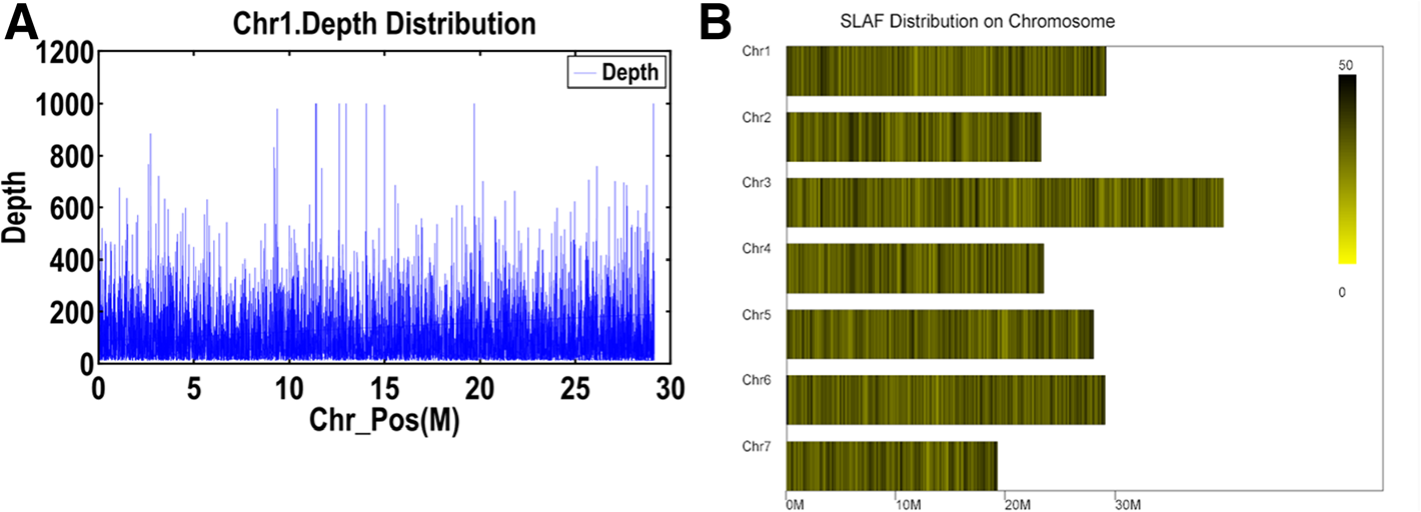
SLAF distribution on chromosome. **a** Depth distribution on chr1; **b** SLAF distribution on each chromosome

By aligning with the cucumber reference genome, all reads mentioned above could be clustered into 73,100 high quality SLAF tags with an average depth of 99.11× (Fig. 2a). According to the genome mapping results, the number of SLAF tags on each chromosome of cucumber was 11,067, 8,799, 14,583, 8,786, 10,565, 11,009 and 7,388, receptivity. Apart from these, a total of 903 tags were located on the scaffolds (See Table 2 and Fig. 2b). Although the number of SLAF tags on each chromosome ranged from 7,388 (Chr7) to 14,583 (Chr3), on the basis of the length of each chromosome, the SLAF tags still evenly distributed on the whole genome indeed, and the average density was about four SLAF tags per 10 kb (Fig. 2b).

**Table 2:** SLAF distribution on chromosome

Chr ID	SLAF number
Chr1	11,067
Chr2	8,799
Chr3	14,583
Chr4	8,786
Chr5	10,565
Chr6	11,009
Chr7	7,388
Other	903
Total	73,100

### Polymorphism analysis

According to the number of alleles and the difference between the SLAF tags, 5,355 polymorphic tags were detected among the 73,100 high-quality SLAFs, with a polymorphism rate of 7.34 %, including 7.09 % SNPs, 0.11 % Restriction Site SNP (RSSNP), and 0.14 % INDEL (Insertion-Deletion). SNPs were the predominant type accounting for 96.6 % of the polymorphism. Apart from this, 92.66 % of the tags were other types, including 91.63 % Non-polymorphism, 0.89 % Unknown type and 0.14 % Repeat (Table 3).

**Table 3:** SLAF type distribution

Type	SNP	RSSNP	INDEL	No Polymorphism	Unknown	Repeat	Total
Number	5,181	79	95	66,985	654	106	73,100
Percent	7.09 %	0.11 %	0.14 %	91.63 %	0.89 %	0.14 %	100 %

**Fig. 3:**
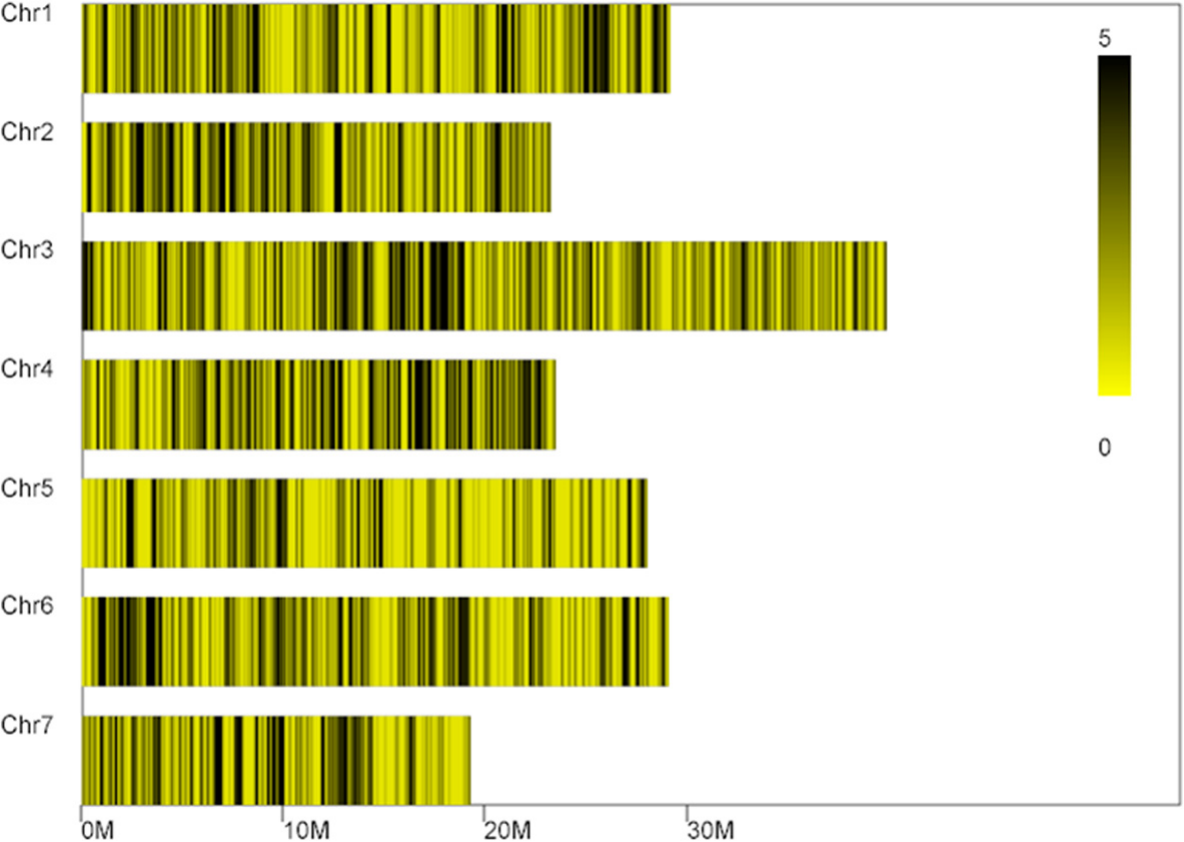
Polymorphic tags distribution on chromosome

The percentages of polymorphic tags were similar to that of SLAF tags for all 7 chromosomes, whereas on the basis of the genomic mapping, 5,355 polymorphic tags were distributed unevenly in each chromosome (Table 4). The number of polymorphic tags on each chromosome of cucumber was 780, 723, 1,145, 765, 544, 817 and 509, receptivity. Also, there were 72 polymorphic tags located on the scaffolds. Take chromosome 1 for example, there was a cluster of tags in the position of 26 Mb (Fig. 3). This may be caused by the germplasm characteristics and the fact that some functional genes are conserved and clustered in genome.

**Table 4:** Marker distribution on chromosome

Chr ID	Marker number
Chr1	780
Chr2	723
Chr3	1,145
Chr4	765
Chr5	544
Chr6	817
Chr7	509
Other	72
Total	5,355

Most of the SNPs were transition type SNPs with Y (T/C) and R (G/A) types accounting for 38.46 % and 27.69 % respectively of all SNPs. The extra four SNP types were transversions including S (G/C), M (A/C), K (G/T), and W (A/T) with percentages ranging from 6.15 to 11.54 % and accounting for 33.85 % of all SNPs. The result also showed us that the ratio between transition and transversion (Ts/Tv) was approximately 1.95:1, which was a little higher than other plants [27].

### Association analysis

**Fig. 4:**
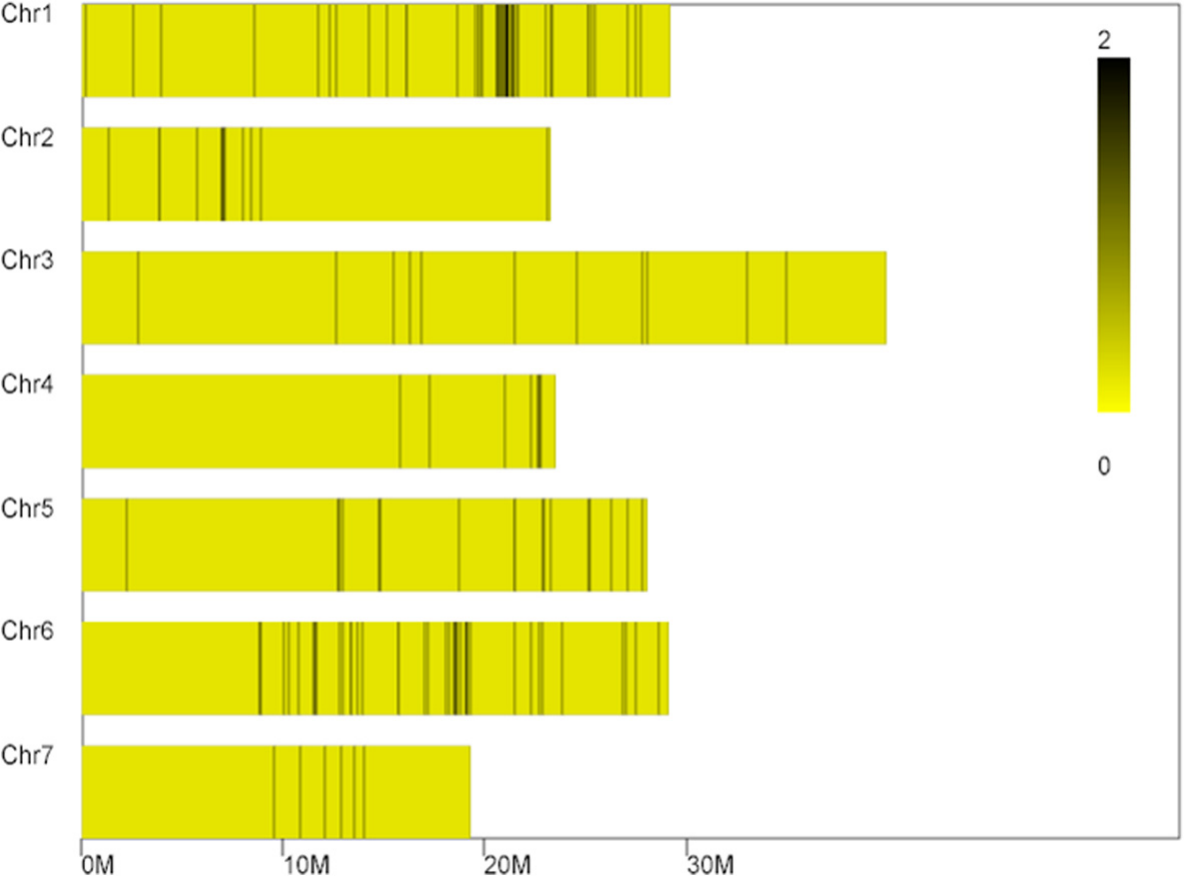
Associated SLAFs distribution on each chromosome

After the standardization, 140 associated SLAFs were identified from the 5,355 polymorphic tags on the basis of Difference Ratio (DR) of each locus in two F_2_ bulks (*Ratio*_*rm* > =1 && *Ratio*_*sm* > =3). As showed in Table 5, the majority of associated SLAFs were distributed in chromosome 1 and 6. The number of associated SLAFs in the two chromosomes was 46 and 39 respectively, which accounting for 60.7 % of the total together (Table 5) (Fig. 4).

**Table 5:** Associated SLAFs distribution on chromosome

Chr ID	Associated SLAFs number
Chr1	46
Chr2	12
Chr3	11
Chr4	7
Chr5	17
Chr6	39
Chr7	6
Scaffold000122	1
Scaffold000143_1	1
Total	140

**Fig. 5:**
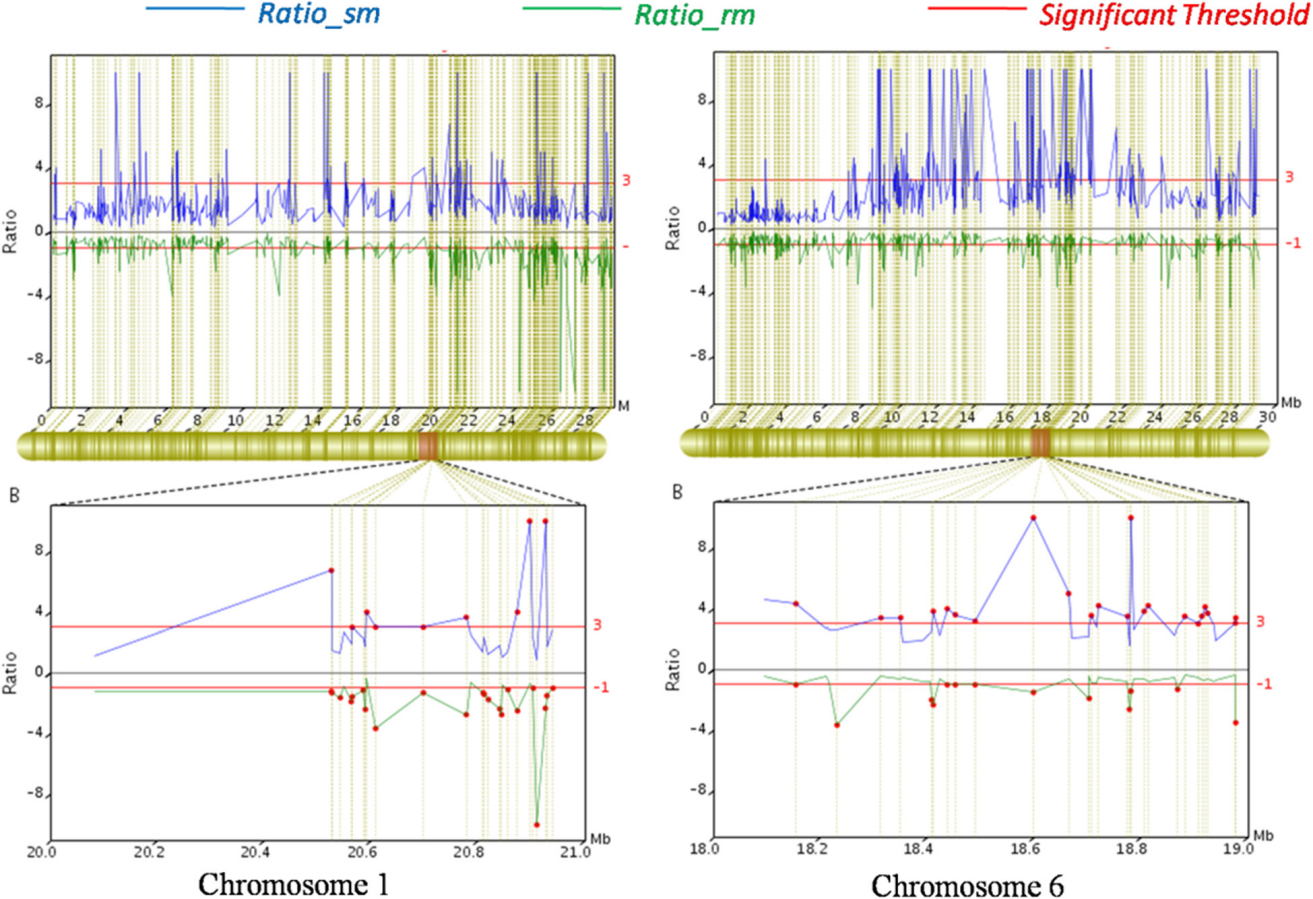
Difference ratio of each locus in Chromosme 1 and 6

According to the distribution of associated SLAFs in each chromosome, we considered the region which involved more than three continuously distributed associated SLAFs as the Hot Region of PMR. As a result, two main Hot Regions were detected on chromosome 1 and 6, which contained three and four continuously distributed associated SLAFs respectively (Fig. 5).

**Fig. 6:**
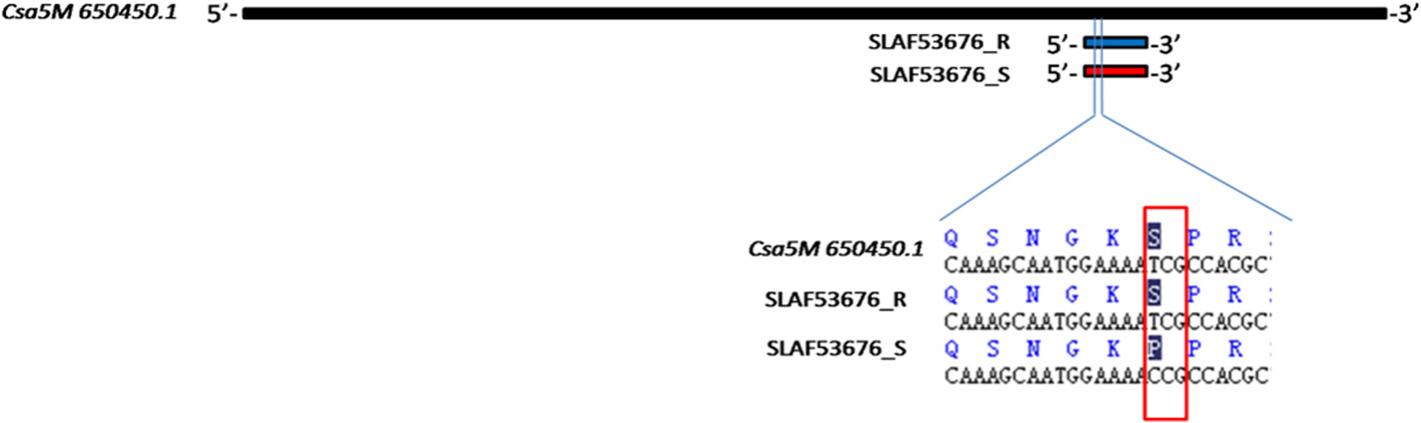
The nsSNP on Csa5M650450.1

Among the 140 associated SLAFs, five SLAFs (SLAF6906, SLAF7937, SLAF30023, SLAF42307 and SLAF53676) derived from the exons of five different genes respectively were identified. Meanwhile, the polymorphism on SLAF53676 was a nonsynonymous SNP (nsSNP), which changed the code of amino acid sequence from Ser to Pro (Fig. 6). Since this mutation would have influences on some biological processes, such as changes of protein structure and function, cell metabolism and gene expression, together with the fact that this SLAF was part of a cyclin-like gene, SLAF53676 was also considered as a target SLAF besides the other five tags.

### Hot region annotation

**Fig. 7:**
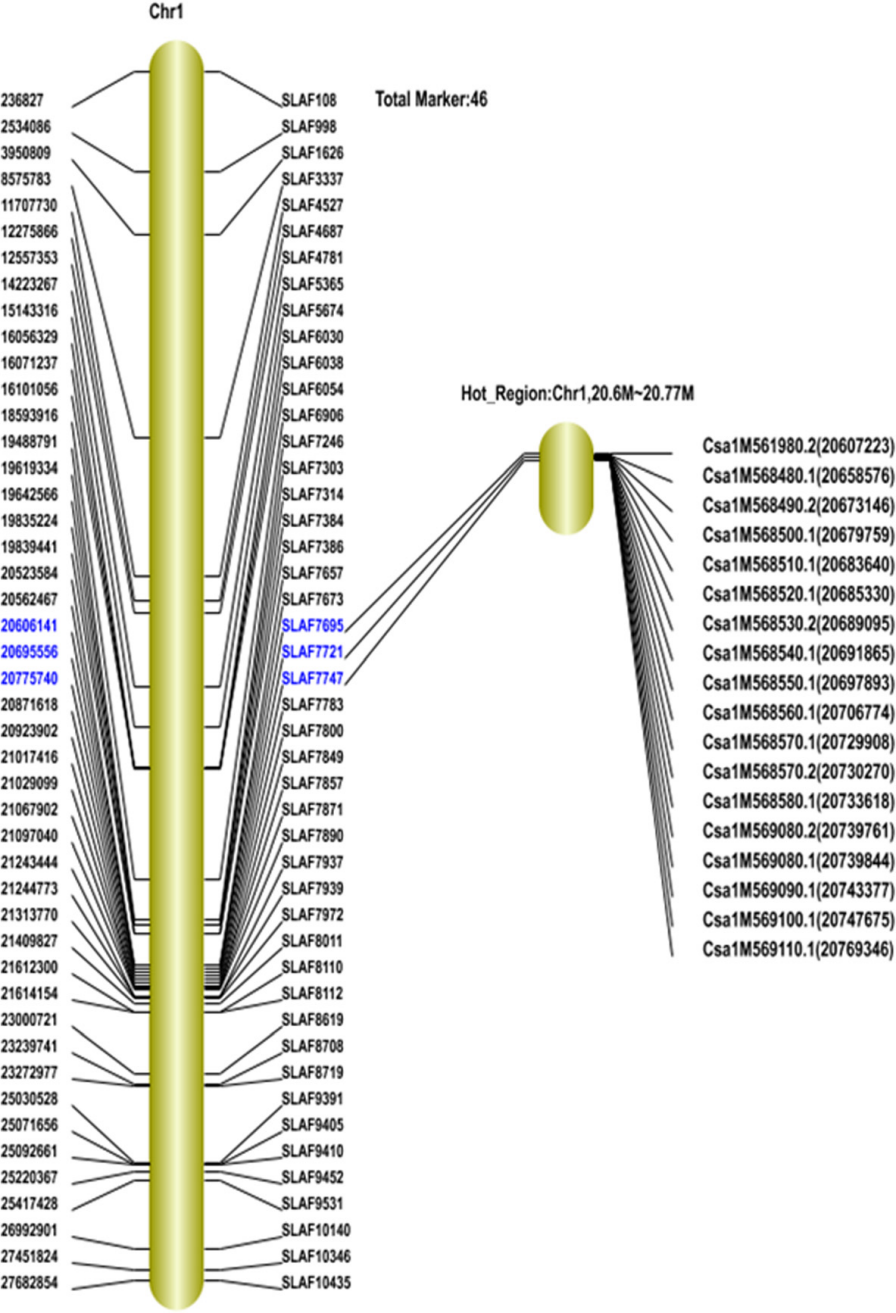
Diff_Markers on chr1

**Fig. 8:**
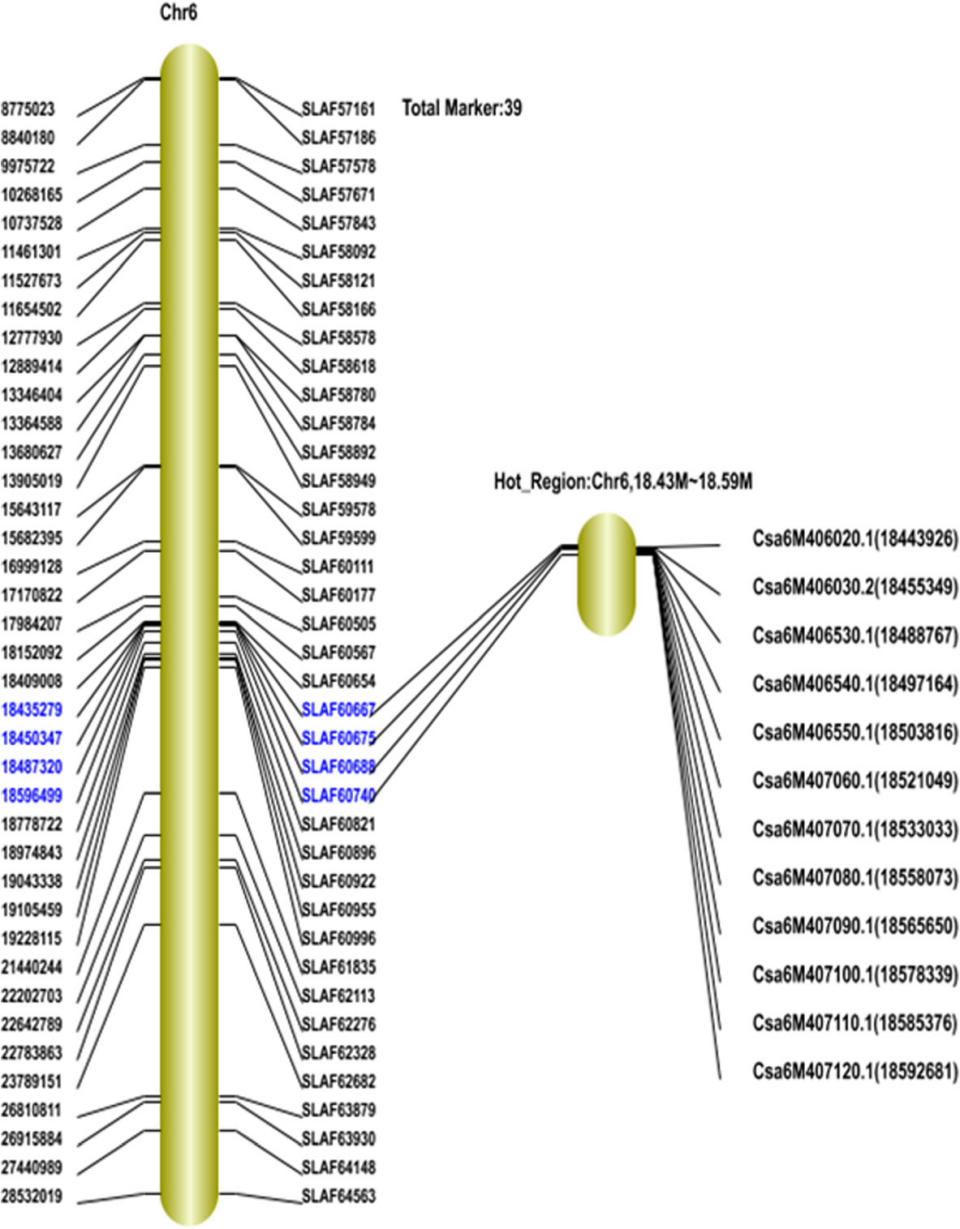
Diff_Markers on chr6

A list of genes within the hot regions was generated using the genome annotation data, after that these genes were used to query the GO annotation on the website of gene ontology to identify putative resistance related genes for further analysis. As a result, a total of 33 putatively genes encoded sequences encompassed in these two regions were used for candidate gene detection by the GO annotation (Table 6) (Figs. 7 and 8). Finally, five genes which related to the defense response, toxin metabolism, cell stress response, and injury response, along with *Csa5M650450.1* were considered as the functional genes associated with PMR in cucumber (Table 7).

**Table 6:** Correlation regional distribution

ChrID	Start	End	Size (Mb)	Diff_marker number	Gene number
Chr1	20,606,000	20,776,000	0.17 M	3	16
Chr6	18,435,000	18,597,000	0.162 M	4	12

**Table 7:** Hot_region annotation

NCBI acc. no	Chromosome/Pos	Biological process	Gene ID	Protein name /Organism
Csa1M568500.1	Chr1,20673146, 20675026,-	defense response (GO:0006952)	F-box protein VBF OS = Arabidopsis thaliana GN = VBF PE = 1 SV = 1	PREDICTED: F-box protein PP2-B15-like [*Cucumis sativus*]
Csa1M568560.1	Chr1,20706774, 20713881,-	cellular response to stimulus (GO:0051716)	Kinesin-4 OS = Arabidopsis thaliana GN = ATK4 PE = 1 SV = 2	hypothetical protein PRUPE_ppa025571mg [*Prunus persica*]
Csa1M569110.1	Chr1,20769346, 20771364,+	defense response (GO:0006952)	Calcium-transporting ATPase 9, plasma membrane-type OS = Arabidopsis thaliana GN = ACA9 PE = 2 SV = 2	PREDICTED: calcium-transporting ATPase 9, plasma membrane-type-like [*Cucumis sativus*]
Csa6M406530.1	Chr6,18488767, 18492584,+	toxin catabolic process (GO:0009407)	Ras-related protein RABF1 OS = Arabidopsis thaliana GN = RABF1 PE = 1 SV = 1	PREDICTED: ras-related protein RABF1-like [*Cucumis sativus*]
Csa6M407080.1	Chr6,18558073, 18563038,+	response to wounding (GO:0009611)	Lysine-specific histone demethylase 1 homolog 3 OS = Arabidopsis thaliana GN = FLD PE = 1 SV = 1	PREDICTED: lysine-specific histone demethylase 1 homolog 3-like [*Cucumis sativus*]

## Discussion

### The advantage of SLAF-seq technology for BSA

SLAF-seq technology has several distinguishing characteristics in contrast to inefficient, expensive, and time-consuming conventional methods of developing markers. Before sequencing, bioinformatics analysis were carried out; the reference genome of cucumber was analyzed, considering the information on genomic GC content, repeat conditions, and genetic characteristics. All these processes above were for designing an efficient marker development approach. The SLAF-seq method also provided us some significant advantages such as developing a large numbers of markers having high accuracy with less sequencing. Overall, the SLAF-seq has the following advantages: i) deep sequencing to ensure genotyping accuracy; ii) reduced representation strategy to reduce sequencing costs; and iii) pre-designed reduced representation scheme to optimize marker efficiency [25].

BSA was developed for rapid identification of markers linked to any specific gene or genomic region [28–30]. The central idea of BSA is to form DNA pools of plants that differentiate with regard to phenotype. Any polymorphic marker with clear differentiation of the two bulks will be closely linked to the respective phenotype. In the present study, the introduction of genotyping by sequencing was a critical development to allow for such a massively parallel approach in a short time to generate enough polymorphic markers for BSA. Therefore, the SLAF-seq based BSA (also called Super-BSA) has the following advantages compared to the traditional BSA: i) large-scale mixed pool, the number of DNA in a pool can reach up to 200 or higher; ii) higher marker density, 100,000 sequenced tags was used for DNA pools scanning; and iii) finer positioning, a large number of genome-wide SNPs were developed.

Compared to the traditional BSA, the greatest advantage of the Super-BSA is the excellent development efficiency. In this study, a total of 73,100 SLAF markers were developed based on high-throughput sequencing, and 5,355 polymorphic tags were generated. Among the 5,355 tags, 140 associated SLAFs related to the PM resistance were identified. While by using the AFLP makers, Jian et al*.* only identified one maker linked with the cucumber PM susceptible gene, and Jing et al*.* also located one closely linked maker by SRAP technique in the other study [31, 32]. In the recent studies, Zhang et al. obtained four loci in their study, as well as that a total of four and six QTLs were successfully validated respectively in different environments [33].

### The confirmation of hot region

**Fig. 9:**
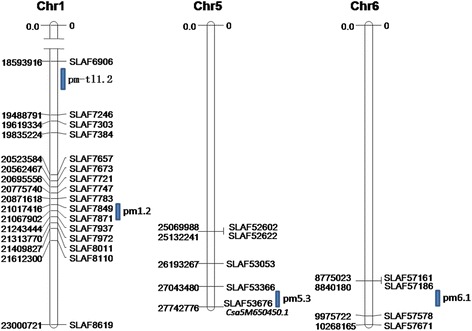
The projection of QTL and associated SLAF on physical map

Several studies have been carried out in cucumber for QTL mapping of PM resistance. These QTL were distributed in six of the seven cucumber chromosomes (1, 3, 4, 5, 6 and 7) with some major QTL (R^2^ > 20 %) mapped in chromosome 1 by Sakata et al. [13], chromosome 5 by Zhang et al. [18] and de Ruiter et al. [14]. In this study, 140 associated SLAFs distributed in all the seven chromosomes were indentified. In order to compare our results with other studies, these SLAFs along with the QTL from other studies on the cucumber were projected on the cucumber physical map together, and the result showed that some loci in this study had happened to be in the same region as the QTL detected in other studies. For example, the hot region in chromosome 1 from the present study was consistent with a major QTL in PI 197088–1 identified by Sakata et al. [13], two QTL (pm1.1 and pm1.2) by Liu et al. [16], one QTL (pm-tl1.2) by He et al. [20], and one QTL (pm1.2) in three different environments by Fukino et al. in chromosome 1 [21]. The gene *Csa5M650450.1* was highly consistent in map location with pm5.3 identified by Fukino et al. in four different environments in chromosome 5. In addition, the hot region identified herein in chromosome 6 was consistent with a minor QTL pm6.1 (R^2^ = 7.0 %), which mapped by Fukino et al. (Fig. 9).

Besides these loci, the other associated SLAFs also deserved for further study. For example, the QTL associated with PMR were mainly mapped on chromosomes 1 and 5, while only a few QTL were on chromosomes 6. In this study, two hot regions on Chr1 and Chr6 respectively were detected. Moreover, an interval (from 9 Mb to 20 Mb) on chromosomes 6 contained a lot number of associated SLAFs, wherein a large part of them had a DR value much higher than the threshold (Fig. 5). This result suggests that some important resistance genes associated with PMR were situated on chromosome 6 in the resistant parent BK2. It was reported that, many plant disease resistance genes are highly conserved in domain, such as: nucleotide-binding site (NBS)-leucine-rich repeat (LRR) class and serine/threonine-protein kinase class etc. which plant NBS-LRR proteins encoded by resistance genes play an important role in the responses of plants to various pathogens, including viruses, bacteria, fungi, and nematodes [34]. Wang et al. analyzed NBS-encoding genes within the whole cucumber genome comprehensively, and found that cucumber has relatively limited NBS-encoding genes. Their study showed that there were a total of 57 NBS-encoding genes on the seven chromosomes, wherein 52 ones clustered on chromosome 2, 3, 4, 5 and 7, and only three and two genes were on chromosome 1 and 6 respectively [35]. Coincidentally, the two NBS-encoding genes on chromosome 6 (*Cucsa.328080* and *Cucsa.102240*) just located in the significant association interval (9 –20 Mb) on chromosome 6. Therefore, in subsequent studies, these two genes should be paid more attention to clarify whether they involve in PMR. If the result proved that they have no contribution, which means that this interval on chromosome 6 must contain other novel genes for PMR. This would be valuable for the diversity expansion of cucumber germplasm resistant to PM.

### The candidate genes for PM resistance

After the GO annotation, five genes related to the defense response, toxin metabolism, cell stress response, and injury response along with *Csa5M650450.1* were considered as candidate genes for PMR.

*Csa1M568500.1* was an F-box protein. The F-box proteins are proteins containing at least one F-box domain, which was a protein structural motif of about 50 amino acids that mediates protein–protein interactions motifs such as leucine-rich repeats (a typical motif of NBS-encoding genes). The F-box proteins have also been associated with cellular functions such as cell cycle transition, signal transduction, gene transcription, male sterility, programmed cell death (PCD) and so on [36]. In plants, many F-box proteins are represented in gene networks broadly regulated by microRNA-mediated gene silencing via RNA interference which are involving in hormone (e.g., ethylene, auxin, gibberellins and jasmonate) signal transduction and biological processes, such as self-incompatibility and floral development [37]. Recent studies also suggested that F-box proteins may be involved in the stress response in plants. Cao et al. identified and cloned defense-related F-box protein gene (*OsDRF1*) in rice, which was suggested to play a role in disease resistance via upregulating defense-related gene expression [38]. Bozkurt et al. cloned a new Zeitlupe (ZTL) type F-box protein gene in barley [39]. In the subsequent study they found that, in response to silencing of this F-box gene via BSMV mediated virus induced gene silencing (VIGS) method, barley plants lost resistance towards avirulent PM race [40]. This observation suggests that F-box protein functions as a positive regulator in PMR reaction.

*Csa1M568560.1* was a kind of kinesins protein, belonging to a class of motor proteins found in eukaryotic cells. Kinesins move along microtubule filaments, and are powered by the hydrolysis of ATP. The active movement of kinesins supports several cellular functions including mitosis, meiosis, transport of cellular cargo, and PCD, which was a biological process regulated by some specific genes in cell growth or response to stimulation outside, along with the characteristics of cell morphology and molecular biology [41]. Plant hypersensitive response (HR) is a type of PCD and also a form of disease resistance induced by the incompatible pathogen with a rapid death of the infected cells and their surroundings to restrict the pathogen growth [42]. The HR progress could also be observed when the cucumber infected by PM. So *Csa1M568560.1* was inferred to be involved in the HR when functioned in the progress of PMR.

*Csa1M569110.1* was predicted to be a Calcium-transporting ATPase, which was a transport protein in the plasma membrane of cells serves to remove calcium (Ca^2+^) from the cell. As a second messenger in plant cell, the changes of Ca^2+^ in concentration could regulate many physiological and biochemical processes, and plays a pivotal role in the growth and development of plants, as well as the reaction and adaptation during environmental changes [43, 44]. In Arabidopsis root cell, Ca^2+^-ATPase in the plasma membrane promote H^+^/Ca^2+^ exchange in order to speed up the Ca^2+^ uptake which maintained the Ca^2+^ concentration in the cell at a normal level and further improved its tolerance to drought. [45] The study in cucumber suggested that application of exogenous calcium ions can increase the mineral absorption and transport, enhanced the activities of ATPase, alleviated the hypoxic injury and enhanced its tolerance to hypoxia [46]. In this study, there was a tightly linkage between the Calcium-transporting ATPase gene and the associated SLAFs, which suggested that this gene may play a certain role in stress response when the cucumber infected by PM, and deserved further study.

By being compared to the database, *Csa6M406530.1* was considered as a Ras-related protein gene. It was a class of small molecules ubiquitous GTP-binding proteins in eukaryotes, which functioned as binary molecular switches for the controlling of intracellular signaling networks, and mainly involved in cell proliferation, signal transduction, endocytosis, vesicular transport, cell growth and differentiation [47]. In medical science, Ras-related protein was highly active in cancer and other diseases, while their functions were rarely reported in the plant research. By using the suppression subtractive hybridization (SSH), a cDNA library of rice leaves induced by sheath blight fungus was constructed, and 63 high quality EST sequences were generated. Functional classification results show that: 50.7 % of the comprehensive analysis EST involved in photosynthesis, and the Ras-related protein may play an important role in rice sheath blight resistance [48]. Besides, the effect of Ras-related protein in plant toxins catabolism was also critica, and these make *Csa6M406530.1* an important candidate gene in cucumber for PMR.

*Csa6M407080.1* was a lysine-specific Histone demethylase 1(LSD1) gene, which encodes a nuclear protein containing a SWIRM domain, a FAD-binding motif, and an amine oxidase domain. This protein is a component of several histone deacetylase complexes, though it silences genes by functioning as a histone demethylase [49]. It’s now known LSD1 complex mediates a coordinated histone modification switch through enzymatic activities as well as histone modification readers in the complex. [49] Dietrich et al. obtained the first Arabidopsis genes *AtLSD1* by map-based cloning methods, and further study showed *AtLSD1* encodes a novel zinc finger protein that negatively regulates plant defense and cell death signaling pathways [50, 51]. In rice, Wang et al. found that overexpression of *OsLSD1* could increase expression of PR-1 mRNA, and an accelerated hypersensitive response when inoculated with avirulent isolates of blast fungus. Both sense and antisense transgenic rice plants conferred significantly enhanced resistance against a virulent isolate of blast fungus. Moreover, ectopic overexpression of *OsLSD1* in transgenic tobacco (Nicotiana tabacum) enhanced the tolerance to fumonisins B1 (FB1), a PCD-eliciting toxin. The above study suggests that *OsLSD1* plays a negative role in regulating plant PCD, and this would be the similar mechanism for *Csa6M407080.1* to participate the cucumber PM resistance [52].

The *Csa5M650450.1* on chromosome 5 was a cyclin-like (cyclin L) gene, which is primary regulators of the activity of cyclin-dependent kinases, and known to play critical roles in controlling eukaryotic cell cycle progression. While there has been extensive research on cell cycle mechanisms and cyclin function in animals and yeasts, only a small number of plant cyclins have been characterized functionally. Recent study implied that the cyclin-like gene in plants may work as an important regulating gene in disease resistance. Xu et al*.* found that the cyclin L homolog *MOS12* and the *MOS4*-associated complex are required for the proper splicing of plant resistance genes [53]. Since was highly consistent in map location with pm5.3, and there was a nsSNP which changed the code of the amino acid sequence from Ser to Pro, the *Csa5M650450.1* was considered as an important candidate gene for PMR.

Associated markers identified by super-BSA in this study, could not only speed up the research on the PMR genes, provide an important way to the marker-assisted PMR cucumber breeding. Moreover, this study could also be extended to any other species with reference genome.

## Conclusion

Based on polymorphic markers developed using the SLAFseq approach, a super-BSA was conducted to identify candidate genes associated with PMR. As a result, two hot regions on Chr1 and Chr6 respectively were detected, and six genes associated with the disease resistance were considered as the candidates for PMR. These genes will be further studied, and the associated SNPs will be used for marker-assisted breeding of PM resistance cucumber.

## Endnotes

Greenhouse and field [1], powdery fungal growth [2, 3], Jinza I, II, III, IV etc. [4], recessive factors [5], 1–2 minor genes [6], PI 200815 or PI 200818 [5], (PI 279465 from Japan) [7], performance of PMR [8], dull fruit color [9–11], cucumber chromosome 5 [12], PMR in cucumber [13–18], MRGH63B in LG V [19], 74.5 % respectively [20], were successfully validated [21], for functional gene mining [22–24], SLAF-seq library [25], using BLAT [26], than other plants [27], or genomic region [28–30], in the other study [31, 32], different environments [33], and nematodes [34], chromosome 1 and 6 respectively [35], death (PCD) and so on [36], and floral development [37], defense-related gene expression [38], protein gene in barley [39], towards avirulent PM race [40], molecular biology [41], restrict the pathogen growth [42], environmental changes [43, 44], tolerance to drought [45], tolerance to hypoxia [46], growth and differentiation [47], heath blight resistance [48], as a histone demethylase [49], signaling pathways [50, 51], PM resistance [52], of plant resistance genes [53], used for subsequent analysis [54], bromide (CTAB) method [55].

## Abbreviations

SLAF: Specific Length Amplified Fragment
PM: Powdery Mildew
NGS: Next-Generation Sequencing
BSA: Bulked Segregant Analysis
PMR: PM Resistance
QTL: Quantitative Trait Loci
RIL: Recombinant Inbred Lines
LGs: Linkage Groups
SNPs: Single Nucleotide Polymorphisms
InDel: Insertion-Deletion
GC: Guanine-Cytosine
RP: Resistant Parent
SP: Susceptible Parent
SM: Susceptible Mix
RM: Resistant Mix
RSSNP: Restriction Site SNP
DR: Difference Ratio
GO: Gene Ontology
DI: Disease Index
CTAB: Cetyltrimethylammonium Bromide
PCD: Programmed Cell Death
